# Effects of tag mass on the physiology and behaviour of common noctule bats

**DOI:** 10.1186/s40462-024-00477-7

**Published:** 2024-05-09

**Authors:** Marit Kelling, Shannon E. Currie, Sara A. Troxell, Christine Reusch, Manuel Roeleke, Uwe Hoffmeister, Tobias Teige, Christian C. Voigt

**Affiliations:** 1 Leibniz Institute for Zoo and Wildlife Research, Alfred-Kowalke-Str. 17, 10315 Berlin, Germany; 2 University of Potsdam, Plant Ecology and Nature Conservation, Potsdam, Germany; 3 School of BioSciences, University of Melbourne, Parksville, 3010 Australia; 4 Natura Büro für Zoologische und Botanische Fachgutachten, Leipzig, Germany; 5 Büro für Faunististische und Ökologische Fachgutachten, Berlin, Germany

**Keywords:** Radio-tracking, Transmitter, Biologging, Energetic, Metabolic power, Body condition, *Nyctalus noctula*

## Abstract

**Background:**

External tags, such as transmitters and loggers, are often used to study bat movements. However, physiological and behavioural effects on bats carrying tags have rarely been investigated, and recommendations on the maximum acceptable tag mass are rather based on rules of thumb than on rigorous scientific assessment.

**Methods:**

We conducted a comprehensive three-step assessment of the potential physiological and behavioural effects of tagging bats, using common noctules *Nyctalus noctula* as a model. First, we examined seasonal changes in body mass. Second, we predicted and then measured potential changes in flight metabolic rate in a wind tunnel. Third, we conducted a meta-analysis of published data to assess effects of different tag masses on the weight and behaviour of bats.

**Results:**

Individual body mass of common noctules varied seasonally by 7.0 ± 2.6 g (range: 0.5–11.5 g). Aerodynamic theory predicted a 26% increase in flight metabolic rate for a common noctule equipped with a 3.8 g tag, equating to 14% of body mass. In a wind tunnel experiment, we could not confirm the predicted increase for tagged bats. Our meta-analysis revealed a weak correlation between tag mass and emergence time and flight duration in wild bats. Interestingly, relative tag mass (3–19% of bat body mass) was not related to body mass loss, but bats lost more body mass the longer tags were attached. Notably, relatively heavy bats lost more mass than conspecifics with a more average body mass index.

**Conclusion:**

Because heavy tags (> 3 g) were generally used for shorter periods of time than lighter tags (~ 1 g), the long-term effects of heavy tags on bats cannot be assessed at this time. Furthermore, the effects of disturbance and resource distribution in the landscape cannot be separated from those of tagging. We recommend that tags weighing 5–10% of a bat’s mass should only be applied for a few days. For longer studies, tags weighing less than 5% of a bat's body mass should be used. To avoid adverse effects on bats, researchers should target individuals with average, rather than peak, body mass indices.

**Supplementary Information:**

The online version contains supplementary material available at 10.1186/s40462-024-00477-7.

## Background

Movement ecology has emerged as an important discipline in behavioural ecology and conservation biology [38, 39], because information on the spatial behaviour of animals provides important insights into habitat use, trophic interactions, seasonal movements, biodiversity patterns and ecosystem functioning [26, 58]. As a result, an increasing number of animals are being tagged with external devices to determine their spatial position at high resolution. In early tracking studies, researchers used high frequency (VHF) radio transmitters to track animal movements [14]. More recently, tags, which rely on the Global Positioning System (GPS), both as loggers and with satellite upload, have become common. New technologies are constantly being developed which can provide location data on free-ranging animals with high spatial and temporal resolution, such as automated high-throughput radio-tracking [40], or collect physiological data such as body temperature [33, 36] and heart rate [44, 61]. Recent technical advances helped to miniaturize tags that can be applied now to even small flying animals [8]. Despite modern lightweight technology, there has been no apparent reduction in relative tag mass (tag mass divided by body mass) for flying animals, even though increasingly smaller animals are being tagged [49]. Nevertheless, the ability of flying animals to carry additional loads is limited [34], and tagging requires consideration of ethical and practical criteria.

Powered flight is an energetically costly form of locomotion [12, 68], and the high energetic demands of flapping flight have shaped the evolution of birds and bats [3, 57]. When comparing different species of birds and bats, aerodynamic theory predicts that flight metabolic rate (metabolic power) scales with body mass by a power of between 0.7 and 1.9, depending on the species investigated [35, 41, 42, 60]. Considering that body mass strongly influences the metabolic rate in flight, a rule of thumb was established by Brander and Cochran [10] that the mass of tags attached to birds should not exceed 5% of their body mass. This critical threshold was based on the assumption that effects on bird behaviour are negligible when tags of this mass or smaller are used. However, empirical support for this suggestion is limited, and some researchers have proposed even stricter thresholds of around 3% [27, 64]. A meta-analysis of the effects of tags on birds found significant negative effects of devices in general, but no evidence of increasing effects with increasing relative tag mass [5]. Specifically, fitness decreased and energy turnover increased in tag-carrying individuals. In birds, the reduced fitness manifested itself in changes in breeding behaviour, as birds tagged during the reproductive period were less likely to breed [5]. Overall, the extent to which the extra load of a tag increases energy expenditure appears to vary greatly between species [5, 64].

Bats and birds differ greatly in morphology and biology, which limits the possibility to extrapolate findings from birds to bats. Apart from the obvious difference between wings covered with feathers and wings formed by thin membranes, bats tend to have a larger wing area than birds of similar size [41]. In addition to wing shape and structure, bats and birds differ in flapping motion, which together affect manoeuvrability [13, 30]. Of the > 1400 extant bat species (Mammal Diversity [18]), the majority feed on insects. Insectivorous bats require high manoeuvrability to capture insects in flight [43]. A reduction in manoeuvrability due to tags carried by bats would be potentially problematic. Indeed, Aldridge and Brigham [1] found that an additional tag load of between 5 and 33% reduced the ability of bats to manoeuvre. Accordingly, they recommend that tag mass remain below a critical threshold of 5% for bats weighing less than 70 g, and that tracking periods should be limited to times when prey is abundant. Since then, numerous studies have used these criteria, yet only a few have aimed at quantifying potential impacts of tagging. Notably, it was confirmed that repeated tagging of the same individuals during subsequent years does not alter the body condition and fitness of some bat species [37]. In addition, it has been proposed that tags exceeding the 5% threshold, e.g. tags of up to 10% of a bat's body mass, should only be applied for a few days [2]. However, some studies have used even heavier tags, equivalent to around 12–14% of a bat's body mass, albeit for relatively short periods of time [17, 20, 51, 53, 69]. Anecdotal evidence suggests that these tags had no negative effects on the health and movements of tagged bats, at least when used for short periods [17]. Voigt and colleagues found no effect of tags on body mass changes when comparing tagged and untagged bats [69]. To our knowledge, no other study has investigated the effects of tags above the 5% threshold on wild bats in a more systematic way. Therefore, we aimed to fill this gap with a comprehensive study by collecting data on natural changes in body mass, conducting experiments in a wind tunnel and performing a meta-analysis of published data. Specifically, we asked whether carrying external loads affects the body condition and behaviour of the common noctule bat *Nyctalus noctula*.

We focused on common noctules because many movement studies have been carried out on this species using tags of different masses [31, 51–56, 62, 69]. As a typical member of the guild of open-space foraging bats [19], the common noctule has a high aspect ratio (wing area in relation to squared wingspan; [41], which allows this bat to move quickly between feeding sites. It has been shown that metabolic rate in flight increases when open-space foraging bats must manoeuvre in small spaces [67], such as forest gaps, illustrating the limited manoeuvrability of these bats due to their high aspect ratio.

In our study, we evaluated the effect of tags on common noctule bats wearing tags of different masses in a three-step approach. First, we assessed how variable body mass is within this species by examining seasonal changes in body mass at the population and individual level. Second, based on aerodynamic theory, we predicted that flight metabolic rate is higher when bats carry tags of more than 10% of the body mass, compared with the untagged condition. We then conducted an experiment with common noctules flying either tagged or untagged in a wind tunnel at 8 m/s. We used the ^13^C-labelled Na-bicarbonate technique to compare flight metabolic rates between treatments [21]. Third, we assessed the potential physiological and behavioural effects of tagging on common noctule bats through a meta-analysis of published literature. This meta-analysis includes data from more than 200 common noctule bats, with relative tag masses varying between 2.7 and 19.4%. We hypothesised that tags would alter the body condition and behaviour of the bats. We predicted that common noctules lose more body mass with increasing tag mass and with increasing deployment duration. Because we assumed a larger impact of heavy tags on bats, we also predicted that bats emerge later and travel shorter periods with increasing tag mass. The results of this study will provide valuable information for deciding how much weight to attach to bats in future tracking studies.

## Methods

### Seasonal body mass changes

*Inter-individual differences in body mass*: We compiled body mass data from common noctule bats (55 monitoring events, n = 3000) regularly surveyed in a colony in southern Brandenburg, Germany (Prieros, 52° 13′ 25″ N, 13° 46′ 19″ E). Individuals from this colony use bat boxes throughout the year, including the hibernation and reproduction period. Most data were collected in July (n = 784). Fewest data were obtained in February (n = 19) and in June (n = 36). No data were collected in January to avoid disturbance in the middle of hibernation.

*Intra-individual differences in body mass*: We also evaluated the seasonal variation of body mass on the individual level by comparing body masses of individual bats throughout the annual cycle; specifically, we looked at the lowest and highest body mass measured for individual bats.

### Flight metabolic rate from aerodynamic theory

To estimate the flight metabolic power of an average common noctule we used the animal flight performance tool, ‘afpt’ package in R [29] (for specific details and input parameters see Electronic Supplement). We first modelled mechanical power across a range of flight speeds for either tagged or untagged common noctules. To account for increased drag associated with carrying a tag, we increased the coefficient of body drag by a conservative 45%, similar to the increase observed in birds carrying comparable devices [48]. As the afpt also takes wingbeat frequency into consideration when calculating mechanical power [28], and wingbeat frequency has been shown to increase with body mass in common noctules [45], we adjusted this coefficient in both models (tagged vs untagged) using the equation of O'Mara et al. [45]. To convert mechanical power output to metabolic power input, we estimated whole-animal conversion efficiency using model predictions from a model of whole-animal conversion efficiency [16]. We then adjusted whole-animal conversion efficiency to muscle conversion efficiency using equation 7 from Hedh et al. [24]. The coefficients input into the afpt are summarised in Table S1 of the electronic supplement.

### Wind tunnel experiment

We used the ^13^C labelled Na-bicarbonate method for measuring metabolic rate of common noctule bats flying at a constant air speed of 8 m/s (see Electronic Supplement for details). For analysis of flight metabolic power, we only used flights where bats flew continuously and steadily, reducing the number of bats from ten to six. Each bat was measured at least once under each flight condition (tagged or untagged). As data were collected over repeated nights for most individuals (up to 3 nights in total), pairwise comparisons were conducted between flight conditions from the same night. Tags were designed to be similar in dimension and mass to GPS units commonly used in past studies. Namely, tags weighed 3.8 g and were 3.1 × 2.0 × 0.7 cm (length × width × height) in size with an attached 5 cm long antennae.

### Meta-analysis of field studies

We compiled data from eight tracking studies of common noctule bats in Germany (see details in electronic supplement, Table S3). Seven of the eight studies used miniaturised GPS loggers with a temporal resolution of 30–60 s; one project used a high-frequency (8 s intervals) and automatized telemetry system called Advanced Tracking and Localization of Animals in a real-life System (ATLAS) (See Table S3 in electronic supplement). A detailed description can be found in Roeleke et al. [56] and Toledo et al. [63]. An important difference is that the data are stored in a remotely accessible database, allowing live tracking, and no tag retrieval is required for ATLAS data collection.

In past studies with GPS loggers, researchers typically recorded the movements of bats over 1–3 nights, so the tags were searched for a few days after tag deployment. Recording started either on the first night, when bats were captured during the day, otherwise on the following night to allow habituation to the tag. If the tags were still attached to the bat, the bat was recaptured to remove the tag. It was therefore possible to obtain body mass data and thus assess changes in body mass.

In our meta-analysis, we included the following variables: tag type, duration of tracking, tag mass, body mass of tagged animal (pre-tagging and at time of recapture), relative tag mass (body-to-tag ratio), emergence time (minutes in relation to sunset), and flight time (min) during the first recorded night. We did not examine the effect of tag mass on travel distances, as this parameter is strongly influenced by the temporal resolution at which spatial positions are collected.

We restricted our analysis of body mass changes to data where tags were actively removed from bats. In total, we included data for body mass at recapture from 149 individuals. For comparison, we also included 15 untagged individuals that were captured and recaptured at the same time (control group). Our data also included unpublished data from malfunctioning loggers that did not yield any tracks, but for which data were available for the corresponding individual. To analyse movement behaviour we pooled these data with data where tags detached independently (i.e., where we do not have body mass data post-tagging) and movement data received remotely from ATLAS tags.

### Data preparation and statistical analyses

Data preparation and analysis were performed using the open-source software R version 4.1.3 [50]. All results are presented as means ± standard deviation. Visualisation, including predictions based on a generalized additive mixed model were performed with the package ‘ggplot2’ [71].

To compare the cost of flight between the tagged and untagged flight conditions we performed a linear mixed effects model with both individual and trial as a random factor. This was chosen to account for the unbalanced design as the number of trials per individual varied between one and three (Table S2, electronic supplement).

To model changes in body mass, emergence time and flight duration, we used generalised linear mixed-effects models (GLMM) fitted with functions *lmer* from the packages ‘lme4’ [7] and function *glmmTMB* from the package ‘glmmTMB’ [11]. Fixed and random effects for each model are listed in Table 1. Model performance was evaluated with the package ‘DARMa’ [23]. We compared models using *model.sel* from the package ‘MuMln’ [6].

**Table 1:** Models of tag mass and additional interacting factors on the physiology and behaviour of bats

Model	Covariates	Estimate	SE	*P*	df
	Impact on body condition				
Recap	Relative change of body mass				
	Intercept	21.22	4.84	< 0.001***	77
	relative tag mass	− 0.003	0.08	0.969	97
	duration of tagging	− 0.686	0.13	< 0.001***	132
	Body mass index (mass/forearm length)	− 44.06	7.42	< 0.001***	135
	Landscape (forest)	1.27	5.87	0.62	6
	Landscape (urban)	0.8	3.66	0.825	4
	Impact on movement behaviour				
Move1	A) emergence time				
	Intercept	3.13	0.34	< 0.001***	
	Relative tag mass	0.03	0.01	0.012*	
	Sex (male)	0.66	0.6	0.274	
	Month	0.11	0.06	0.052	
	Landscape (forest)	− 0.44	0.25	0.082	
	Landscape (urban)	− 0.62	0.18	< 0.001***	
	Sex (male): month	− 0.05	0.08	0.52	
Move2	B) flight duration				
	Intercept	3.48	0.37	< 0.001***	108
	Relative tag mass	0.08	0.01	< 0.001***	48
	Sex (male)	− 2.49	0.79	0.002**	116
	Month	− 0.02	0.06	0.748	114
	Landscape (forest)	0.4	0.27	0.192	6
	Landscape (urban)	− 1.08	0.22	< 0.001***	32
	Sex (male): month	0.33	0.11	0.005**	116

All models included year as random effects (formula: ~ landscape | year, groups: year n = 7). df = degree of freedom, significance codes: ***< 0.001, **< 0.01, *< 0.05, not significant

## Results

### Natural variation in body mass of common noctule bats

**Fig. 1:**
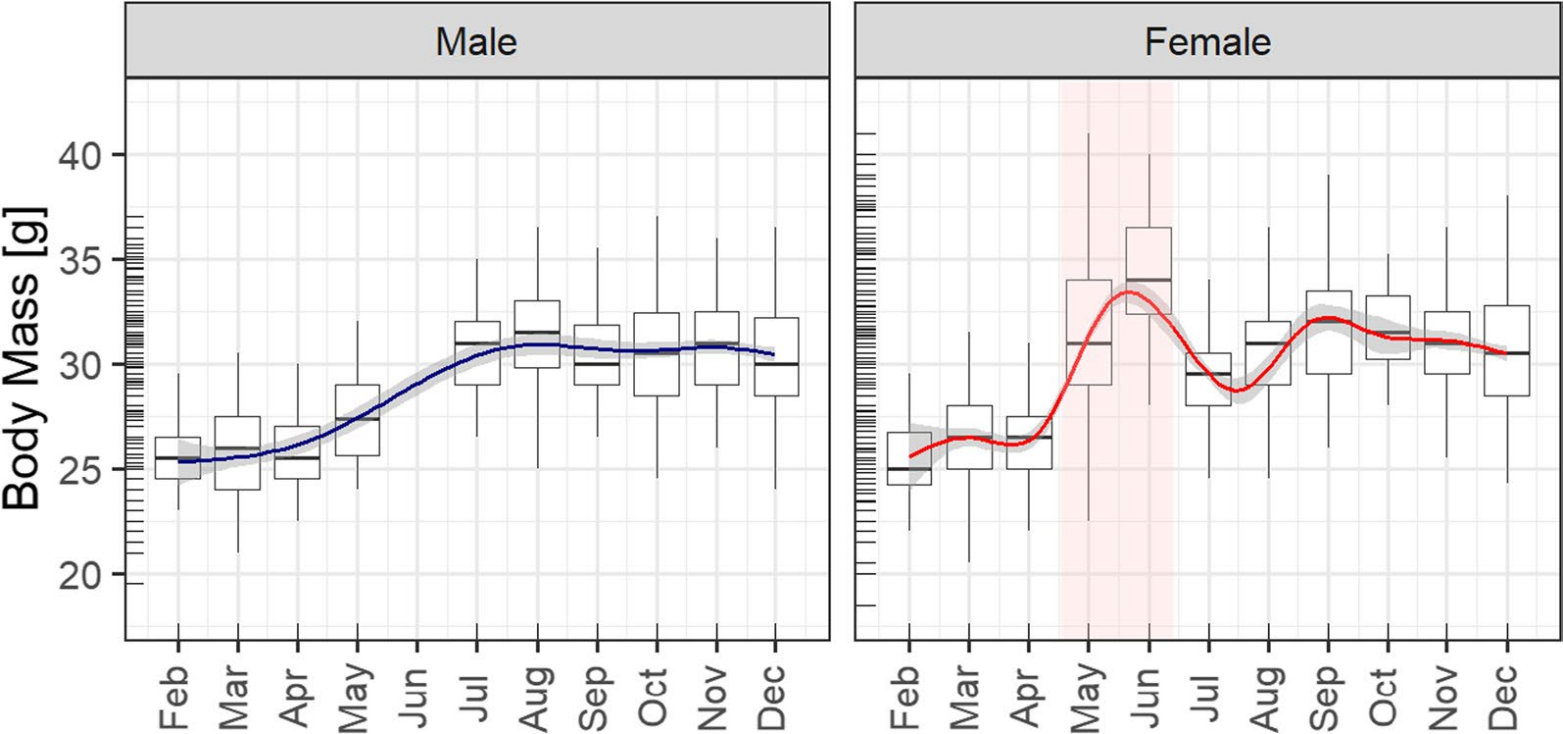
Seasonal variation in body masses of 747 male and 1265 female common noctule bats *N. noctula* from a colony in Northeast Germany. Box plots show adult bat body mass in a given month, coloured background shows pregnancy period. Rug plots on the y-axis show the distribution of individual body masses. Solid line curves indicate the predicted mean with grey area as 0.95 confidence interval from generalized additive mixed model; outliers excluded. No data were collected in January to avoid disturbance in the middle of hibernation

In total, we analysed body parameters from 1139 individual bats, covering a 10-year period. Body mass increased during the spring, starting from the lowest values during hibernation in February. Patterns of body mass changes diverged between female and male common noctule bats in late spring and in early summer (Fig. 1).

Female body mass was lowest in February with a mean of 25.6 ± 2.4 g (range: 22.0–29.5 g). Thereafter, the body mass of females increased until early summer (June: mean = 34.3 ± 2.9 g, range: 28.0–40.0 g). The peak body mass of females coincided with late pregnancy in June. After parturition, body masses decreased to 29.3 ± 2.2 g in July (range: 18.5–38.0 g). In September, body mass of females showed a second peak (mean = 31.9 g ± 2.8 g, range: 26.0–39.0 g). Overall, female body mass averaged 29.8 ± 3.4 g (range: 18.5–41.0 g) throughout the full annual cycle. Body mass index (ratio of mass/forearm length) for females averaged 0.55 ± 0.14 g/mm (range: 0.35–0.78 g/mm).

Body mass of males was lowest in hibernating individuals towards the end of winter, with an average of 25.7 ± 2.0 g (range: 23.0–29.5 g) in February. After the end of the hibernation period, body mass of males increased during spring and early summer, reaching an average peak value of 31.6 ± 2.5 g (range: 26.5–36.5 g) in August. During the full annual cycle, body mass of males averaged 29.5 ± 3.2 g (range: 19.5–37.0 g). Body mass index for males averaged 0.55 ± 0.07 g/mm (range: 0.37–0.76 g/mm). From the onset of hibernation in November, the body mass of both sexes decreased towards the end of the year.

On an individual level, we compared lowest body masses of individuals after the hibernation period (February or March) with those of the same individuals in late summer (August or September), when body mass peaked. Data from 18 common noctule bats for which we obtained data from both seasons showed that body mass changed on average by 7.0 ± 2.6 g (range: 0.5–11.5 g), which is an average gain in body mass of 26.5 ± 11.7% (range: 1.8–54.8%) following the hibernation period. This corresponds to an average change in body mass index of 0.13 ± 0.05 g/mm (range: 0.01–0.22 g/mm).

### Tag mass effects on flight metabolic rate based on aerodynamic theory

**Fig. 2:**
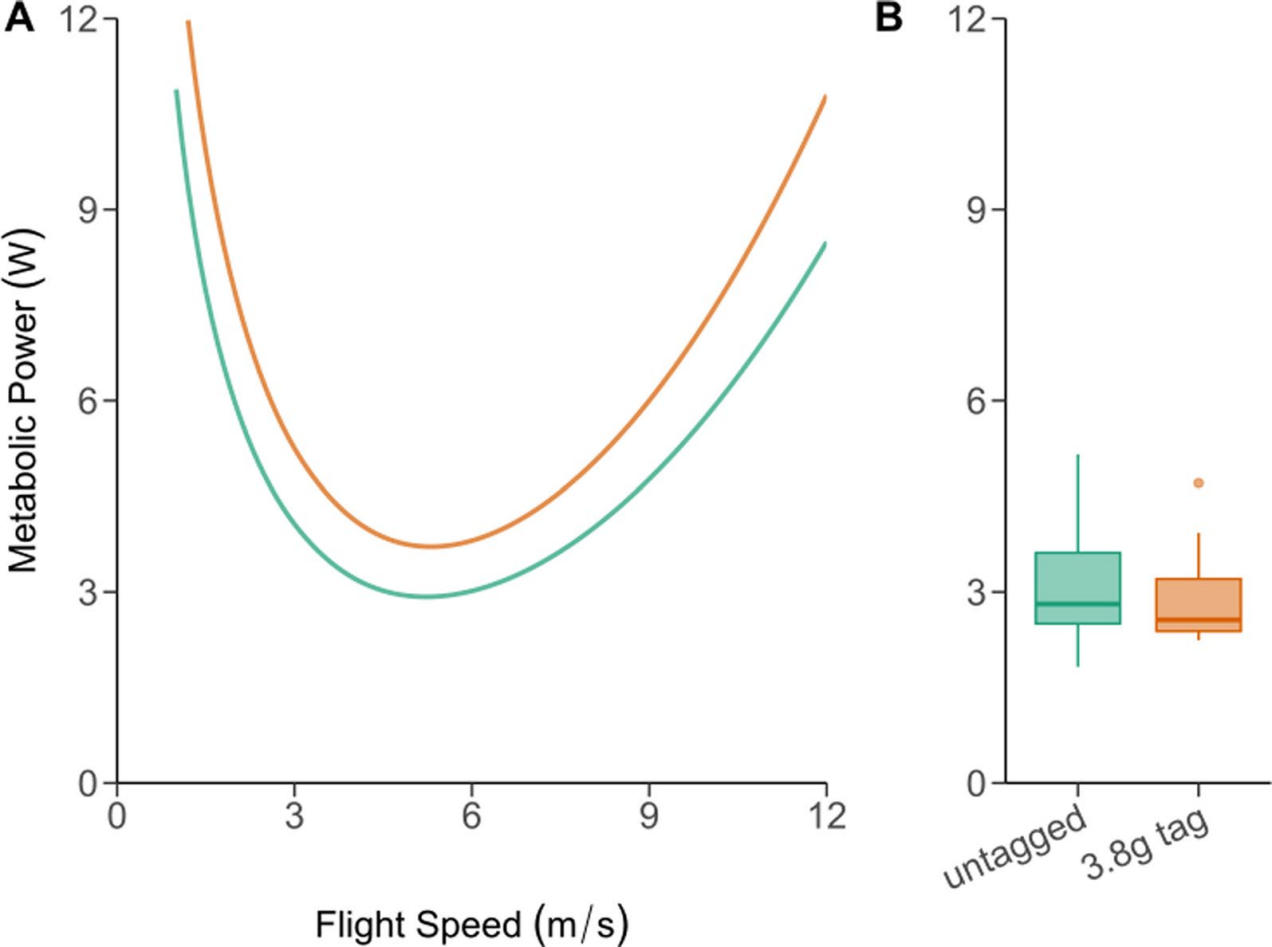
**A** Metabolic power, as predicted by the afpt, of an average common noctule bat across a range of flight speeds (green line) in comparison to the same theoretical bat carrying a 3.8 g GPS tag (orange line). **B** Box plots (25th and 75th percentiles) show measured metabolic power of six common noctule bats flying in a wind tunnel at 8 m/s, either with or without a 3.8 g GPS tag

Using the animal flight performance tool, we predicted the metabolic power of an average untagged common noctule to be 3.96 W when flying at 8 m/s. Whereas when carrying an additional 3.8 g tag, equating to 14% of body mass, and accounting for increased drag, the predicted metabolic power increased to 4.97 W at the same flight speed—a predicted 26% increase in flight metabolic rate (Fig. 2A).

### Wind tunnel experiments

**Fig. 3:**
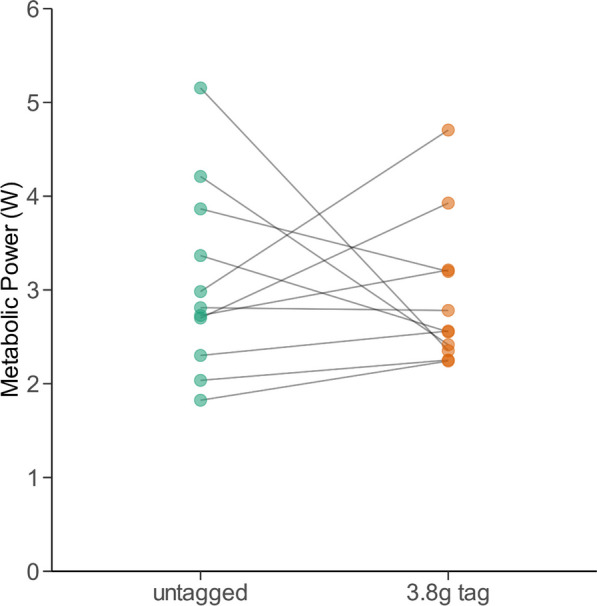
Flight metabolic power of six common noctule bats (n = 22 flight trails) flying in a wind tunnel at 8 m/s, either with or without a 3.8 g GPS tag. Metabolic costs of flight increased in six of 11 flight trials, compared to four where costs decreased and one where costs were similar

Metabolic power in flight averaged 3.1 ± 1.0 W under control conditions (range: 1.8–5.2 W), slightly lower than predicted from aerodynamic theory. Overall, we did not observe a significant difference in flight metabolic power when bats carried GPS tags (mean 2.9 ± 0.8 W; lmerTest, t_11_ = − 0.364, *p* = 0.72) (Fig. 2B). In fact, in 36% of the flight trials we measured a lower metabolic power in bats carrying additional mass. When metabolic power was higher in bats flying with GPS tags, the proportionate increase in cost varied between 11 and 58%, with a mean of 28% (Figs. 2B, 3).

### Meta analysis of movement data from wild bats

Our literature review revealed published studies with a total of 149 common noctule bats that were recaptured and weighed twice, including 134 tagged and 15 untagged bats. Overall, none of the studies explicitly reported on injuries after tag removal or animals recaptured without tags. On average, tags remained on bats for 4 days (range: 1–16 days). In 75 cases, the total duration of tag deployment remained unknown. In seven out of eight studies, the tags were retrieved to download the tracking data from the GPS loggers, while in one study the data were accessed remotely. Body mass of untagged bats (n = 15) was measured a second time after an average of 5 days (range: 1–13 days).

**Fig. 4:**
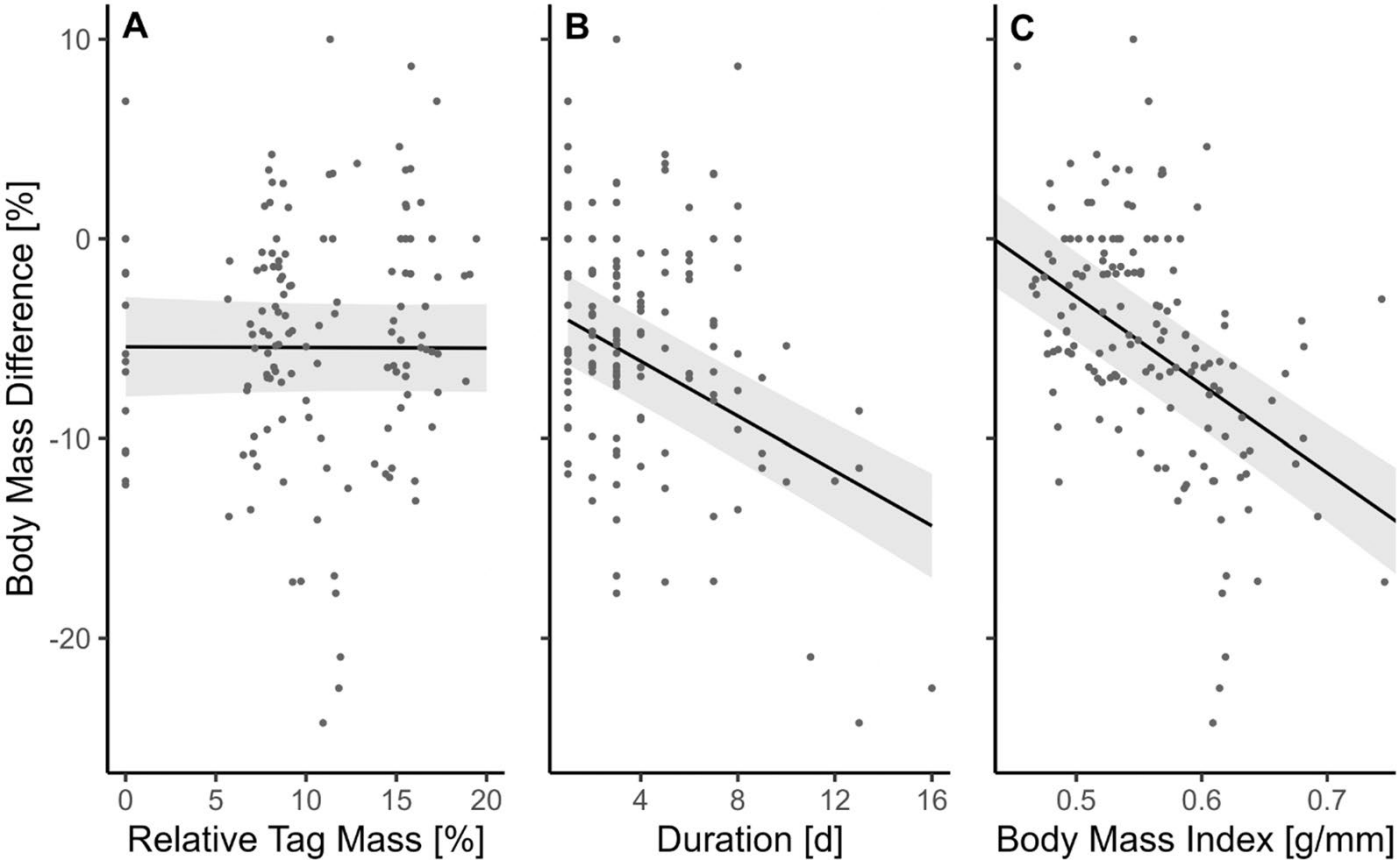
Predicted changes in body mass of common noctule bats (means ± 95% confidence intervals) between tagging event and tag removal, in relation to relative tag mass (**A**), tagging duration in days (**B**) and body mass index in g/mm (mass/forearm) (**C**). All other covariates of the model were kept constant at their mean values. Points show measurements of 149 individuals collected during eight tracking studies (Table S3)

Body mass of bats decreased with increasing duration of tag deployment (− 0.69% each day, *p* < 0.001; Table 1; Fig. 4B), yet this body mass decline was independent of the relative tag mass (− 0.003% for 1% increase in tag mass, *p* = 0.97; Fig. 4A). On average, tagged bats lost 0.5 ± 0.8 g (range: − 4 g to + 2 g) per day during the tracking period, representing on average a daily body mass loss of 1.6% of the initial mass (range: − 11.8 to + 6.9%). Untagged bats lost on average 1.5 ± 1.9 g (range: − 4 g to + 2 g, equivalent to 4.9% of the body mass (range: − 12.3 to + 6.9%). Body mass index at the time of tagging had the greatest negative effect on the changes in body mass after tag deployment, i.e., the higher the body mass index, the more body mass a tagged bat lost (− 4.4% for each 0.1 g/mm, *p* < 0.001; Fig. 4C). The body mass of tagged bats was affected similarly regardless of sex, month and reproductive status (interaction of sex and month), therefore these factors were excluded in the model of their physical state (Table 1, model “Recap”).

**Fig. 5:**
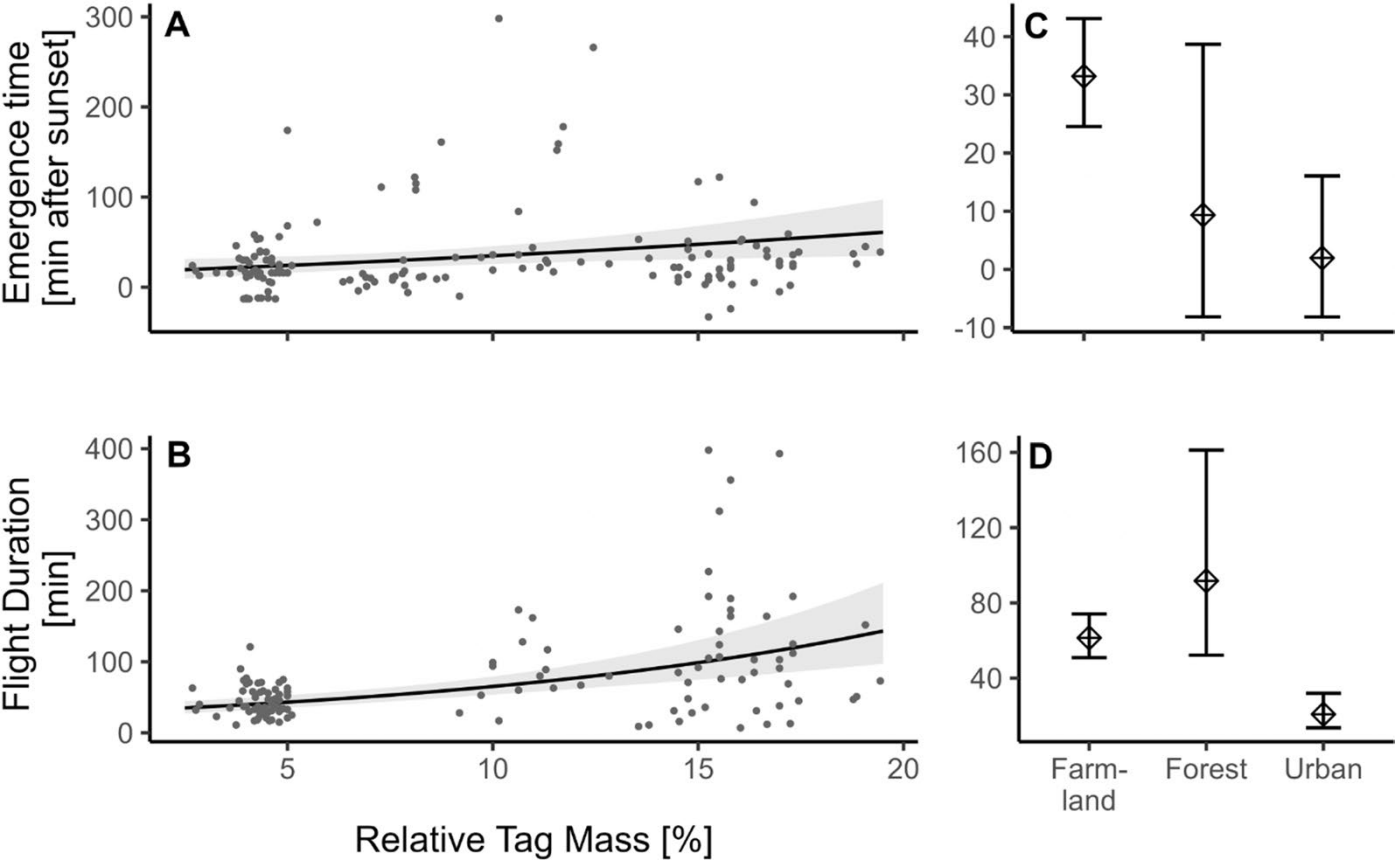
Predicted emergence time (**A**) and flight duration (**B**) with increasing relative tag mass. Right panel shows predicted emergence time (**C**) and flight duration (**D**) categorised by dominant landscape type. All other model covariates were kept constant at their mean values. Points show measurements of 158 individuals collected during eight tracking studies (Table S3)

We examined tag mass effects on emergence time and flight duration in 158 individuals. On average, bats emerged from their colony 36 ± 54 min after sunset (range: 33 min before—360 min after sunset). Total flight duration averaged 75 ± 71 min (range: 7–398 min) during the first recorded night of tagging. Individual analyses indicated that the tags affected the emergence time and flight duration towards later emergence and longer trips (Fig. 5; Table 1).

## Discussion

Tagging bats with loggers or radio transmitters is important for understanding the movement ecology of individuals, but it may affect their behaviour and ultimately body condition. Here, we studied the effects of tagging on flight metabolic rate, body mass and behaviour of bats using the common noctule bat *Nyctalus noctula* as a model.

Similar to other temperate zone bats, body masses of common noctules varied seasonally with the lowest body mass recorded at the end of hibernation and highest body mass recorded in summer [4, 70]. The average body mass fluctuation of individuals recorded in both season was ~ 7 g, demonstrating that bats can experience a 25% change in body mass over the course of a year. Accordingly, the flight metabolic rate of common noctule bats may vary substantially over time, as the intra-specific allometry of flight metabolic rate increases more strongly with increasing body mass than the inter-specific allometry [41, 46, 66].

Based on the aerodynamic model of Klein Heerenbrink et al. [28], we predicted an average increase in flight metabolic rate of 25.9 ± 0.3% for a common noctule bat tagged with a 3.8 g logger flying at wind speeds of 6–10 m/s. In our wind tunnel experiment, the flight metabolic rate of untagged common noctule bats averaged 3.1 W when flying close to minimum power speed. This value is similar to the prediction for a 28 g bat based on simple allometry (3.2 W, [72]), but less than predicted from our aerodynamic model (3.9 W). However, attaching 3.8 g tags to common noctule bats did not result in the expected overall increase in flight metabolic rate of 26%. This finding is surprising given that previous studies in birds have found increases of ~ 5% in flight metabolic rate in birds carrying tags equivalent to ~ 3% of their body mass [64]. While we did detect increases in metabolic rate in some individuals, this result was not necessarily consistent within individuals or across different flights. Other studies in birds have also produced variable results, which supported no strong increases in metabolic rate when flying with additional loads [22, 59]. As the ^13^C-labelled Na-bicarbonate method has been shown to resolve even small changes in flight metabolism at various species in the past [15, 67], we rule out that this method was too imprecise to detect the expected difference. Possibly, the flight behaviour of tagged and untagged noctules was too variable to allow us to resolve a tag mass effect, even though we followed a conservative approach by using only data of bats that flew without major manoeuvres. Small changes in flight speed during the short flight trials is something we cannot directly control for and this may have impacted our findings. Since the wind tunnel was designed for flying vertebrates with a maximum wing span of 0.80 m [47], we reject the idea that the flight chamber obstructed the flight of bats. We conclude that the metabolic rates of tagged noctule bats do not increase much, if at all, compared to the untagged condition, potentially due to slight changes in the wing beat kinematics or compensations in flight. Therefore, future research should examine potential changes in wing beat kinematics and power output using, e.g., acceleration sensors and tomographical particle image velocimetry [16].

Our meta-analysis revealed that most individuals lost body mass after the tagging. Interestingly, body mass losses did not vary with tag mass but with the duration of tag deployment and initial body mass index. The dataset from a previous study revealed body mass losses in both tagged and untagged bats [69], however, it is important to acknowledge the low sample size for untagged bats. The consistent body mass loss of tagged and untagged bats is at least partially caused by the fact that initially bats are captured during the first half of a day for tagging, but often they are recaptured in the evening when emerging from an alternative roost. Previous studies have shown that daily changes in body mass can amount to about 10–40% of the body mass of bats [65, 72]. Likely, common noctules show similar daily body mass fluctuations.

Alternatively, disturbance and handling can also contribute to body mass loss. For example, disturbance of daytime roosts often results in tagged bats moving to other roosts (pers. observation). Possibly, an increased activity caused by roost exploration and social interaction with unknown conspecifics may lower foraging periods of disturbed bats, which may eventually lead to body mass losses. We plead for future studies to be conducted where body mass is measured at the same time of day in both tagged and untagged bats in order to separate disturbance from tagging effects.

We also studied whether tagging of bats affected the emergence and flight duration in common noctule bats. Overall, the recorded emergence times were similar to those previously reported for common noctule bats [25]. While emergence time and flight duration differs between landscapes, our data supports the trend of a delayed emergence and extended foraging trips with increasing tag mass. We speculate that tagged bats may prefer a safer environment with respect to foraging due to the unfamiliar additional load; i.e., tagged bats emerging late may be better protected from visually oriented predators such as owls [32], but they may lose foraging opportunities because insect abundance peaks around sunset. Also, tagged bats may perform longer foraging trips to compensate for the later emergence, or increased energy requirements because of the added tag mass, constrained manoeuvrability and thus impaired hunting success [1]. Furthermore, tagged common noctules emerging late from roosts may lose the benefits of hunting in a group [56]. That said, we note that our dataset is limited to the first night, because in most cases GPS tags recorded the activity of bats only for a single night. Therefore, we do not know whether bats may get used to the extra load of tags. In future, technological advances may facilitate the recording of multiple nights, enabling the investigation of habituation effects in more detail.

Our study is the first to evaluate the potential impacts of tags on the body condition and behaviour of a bat species, covering theoretical models, empirical data and ranging from individual experiments to a meta-analysis. Although comprehensive, we acknowledge that our approach has limitations. For example, our meta-analysis is based on data collected from free-ranging bats. Consequently, observed differences between studies are of a correlational nature. Therefore, we can only speculate for or against tag mass and deployment effects. In particular, we were not able to disentangle the effects of tag mass from the effects caused by the landscape in which bats were studied. We recommend tagging bats with different tag sizes in the same landscape and within the same season. Since we could not tag bats without disturbance, it will be important for future studies to distinguish between the effect of disturbance, including both roost disturbance and bat handling, compared to the effect of the tag itself. All studies included in our meta-analysis aimed to reduce the impact on bats by limiting the recording period to a few days. Therefore, we cannot report on how the behaviour of bats might have changed over an extended period of time, i.e. whether bats might have become used to carrying the additional mass of a tag, or whether the condition and health of bats would deteriorate over time. The observation of a steady decline in body mass with the duration of tag deployment argues against a habituation. In addition, we did not consider the length, width and height of tags as an additional factor in our analysis. Past studies have shown that tag dimensions may as well affect flight performance of aerial vertebrates [9, 64]. Future experimental work in wind tunnels should focus on optimising the shape of the tags to minimise drag.

## Conclusion

Common noctule bats are physically capable of carrying relatively heavy tags, but a combined effect of disturbance and tag deployment could affect their behaviour and foraging success. In our study, we could not separate these effects, as both tagged and untagged bats lost body mass over time. In this species, extended tagging over several days and higher initial body mass index appear to result in greater body mass loss. Bats with low body mass index seem to be more resilient to body mass losses after tagging. Our analysis does not suggest a critical upper threshold of relative tag mass, but since tags were mostly below 10%, and we could not reveal an effect of tag mass on body mass, we recommend keeping relative tag mass below 10% of a bat's body mass and ensuring that tag deployments are limited to a few days for heavy tags. We recognise that this recommendation may not apply to all bat species and tag types, as bats vary widely in size and wing morphology, and tags vary in size and the way they are attached to bats. Since we cannot rule out that heavier tags can affect behaviour, long-term tracking studies should establish a 5% threshold for tag application. We assumed that common noctule bats would be highly vulnerable to tag mass effects due to their high aspect ratio. Bats of other foraging guilds, especially those with a low aspect ratio, may be better able to carry tags. We therefore call for species- or guild-specific studies to assess the potential role of tag mass on body condition and behaviour of bats.

### Supplementary Information

Supplementary file 1: Detailed method description: Aerodynamic model. Figure S1: Measurement example using ImageJ to get wing area and wingspan. Measurements were multiplied to get tip to tip wing span and full wing area. Table S1: Coefficients input into the animal flight performance tool (afpt) averaged from 24 male common noctule bats in Brandenburg, Germany. Detailed method description: The ^13^C labeled Na-bicarbonate method. Figure S2: Correlation between CO2 production rate during the pre-flight period measured by conventional respirometry, i.e. CO2 analyser, and by the ^13^C labelled Na-bicarbonate method (NABI). Table S2: Number of trials per individual common noctule bat flying in the wind tunnel. Table S3: Master table of all tracking studies included in the meta-analysis.

## Data Availability

Data and code are stored in a GitHub repository at https://github.com/maritkelling/tag_mass_nnoc.git.
