# Supplementary material for: Effects of tag mass on the physiology and behaviour of common noctule bats
Source: Mov Ecol. 2024 May 9;12:38. doi: 10.1186/s40462-024-00477-7 (PMC11084088; doi:10.1186/s40462-024-00477-7)
Supplement: Supplementary file 1 — Detailed method description: Aerodynamic model. Figure S1: Measurement example using ImageJ to get wing area and wingspan. Measurements were multiplied to get tip to tip wing span and full wing area. Table S1: Coefficients input into the animal flight performance tool (afpt) averaged from 24 male common noctule bats in Brandenburg, Germany. Detailed method description: The 13C labeled Na-bicarbonate method. Figure S2: Correlation between CO2 production rate during the pre-flight period measured by conventional respirometry, i.e. CO2 analyser, and by the 13C labelled Na-bicarbonate method (NABI). Table S2: Number of trials per individual common noctule bat flying in the wind tunnel. Table S3: Master table of all tracking studies included in the meta-analysis. [file 40462_2024_477_MOESM1_ESM.docx]

ELECTRONIC SUPPLEMENT

**Aerodynamic model**

For the aerodynamic models, we used the animal flight performance tool from the R package ‘aftp’, which has been shown to perform well in estimating mechanical power output in other bat species (Currie et al., 2023; Håkansson et al., 2017). This model enables power calculations for animals carrying a load of specified mass. Morphological characteristics and body mass for an average common noctule were collected from 24 adult or full-grown subadult males, sampled in September 2023 from the same colony as described before. Although we only measured male bats for flight cost calculations, we consider sex bias to be negligible as there are no sex differences in wing shape (O’Mara et al., 2016). We took photos of the bats with one wing outstretched on graph paper and then calculated wing area and wing span (tip to tip) using ImageJ (Schneider et al., 2012) (FIGURE S1) following Pennycuick (2008).

**References:**

Håkansson, J., Jakobsen, L., Hedenström, A., & Johansson, L. C. (2017). Body lift, drag and power are relatively higher in large-eared than in small-eared bat species. *Journal of The Royal Society Interface*, *14*(135), 20170455. <https://doi.org/doi:10.1098/rsif.2017.0455>

O’Mara, M. T., Bauer, K., Blank, D., Baldwin, J. W., & Dechmann, D. K. N. (2016). Common Noctule Bats Are Sexually Dimorphic in Migratory Behaviour and Body Size but Not Wing Shape. *PLOS ONE*, *11*(11), e0167027. <https://doi.org/10.1371/journal.pone.0167027>

Pennycuick, C. J. (2008). Chapter 1 Background to the Model. In *Theoretical Ecology Series* (Vol. 5, pp. 1-20). Academic Press. <https://doi.org/https://doi.org/10.1016/S1875-306X(08)00001-4>

Schneider, C. A., Rasband, W. S., & Eliceiri, K. W. (2012). NIH Image to ImageJ: 25 years of image analysis. *Nature Methods*, *9*(7), 671-675. <https://doi.org/10.1038/nmeth.2089>


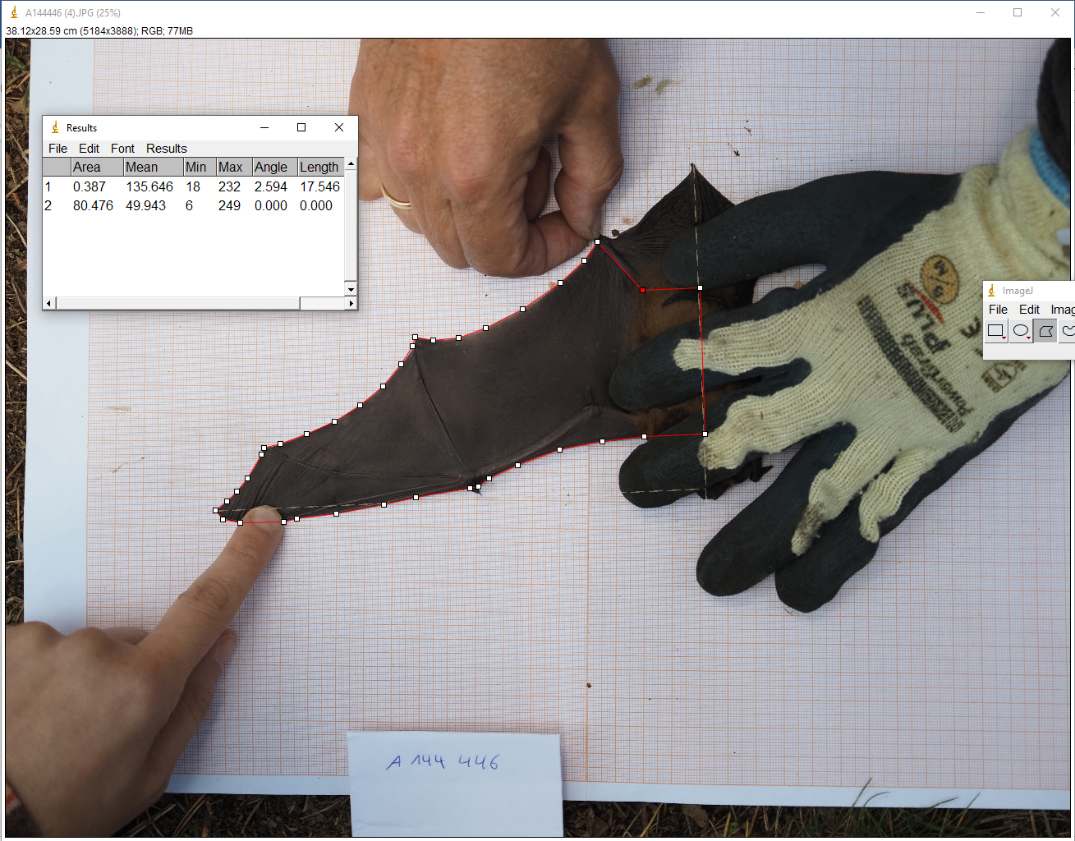


FIGURE S1 Measurement example using ImageJ to get wing area and wingspan. Measurements were multiplied to get tip to tip wing span and full wing area.

TABLE S1 Coefficients input into the animal flight performance tool (afpt) averaged from 24 male common noctule bats in Brandenburg, Germany.

| Treatment type | Mass empty (kg) | Mass load (kg) | Coefficient of body drag (Cdb) | Wing span (m) | Aspect ratio | Wing area (m^2^) | Conversion efficiency (η) | Wingbeat frequency (Hz) |
| --- | --- | --- | --- | --- | --- | --- | --- | --- |
| Untagged | 0.028 | 0 | 0.4 | 0.362 | 8.035 | 0.0163 | 0.087 | 7.52 |
| Tagged | 0.028 | 0.0035 | 0.58 | 0.362 | 8.035 | 0.0163 | 0.087 | 7.76 |

**The ^13^C labeled Na bicarbonate method**

Experiments were conducted in the wind tunnel facility of the Max-Planck Institute for Ornithology in Seewiesen under licence 55.2–1–54–2532–12–2014 granted by the federal country of Oberbayern, Germany. Details related to the closed-circuit wind tunnel at Seewiesen have been previously described by Engel et al. (2006). We obtained 10 adult male common noctule bats in September 2015 from bat boxes in the Berlin area under licence I E229-OA-AS/G/1051 from the ‘Senatsverwaltung für Stadtentwicklung und Umwelt’ in Berlin. Following a period of training, we performed experiments with each individual with and without a 3.8 g GPS tag (Vesper, ASD Technology, Haifa, Israel). For all experiments with common noctule bats, we used the expected minimum power speed of 8 m/s.

For measuring the metabolic rates of flying bats, we used the ^13^C-labelled sodium bicarbonate method (Hambly et al., 2002; Hambly & Voigt, 2011). At the start of each experiment, a bat was released into the wind tunnel for a warm-up flight of approximately 10 min. Individuals were then placed in a 1.3 L respirometry chamber through which CO_2_ free air passed at a constant rate of 1.2 L min^−1^ controlled via a mass-flow controller (MFS-2, Sable Systems, Las Vegas, NV, USA). We monitored ^13^CO_2_ and ^12^CO_2_ enrichment in the outlet air of the chamber using a cavity ring-down spectrometer (G1101 CO_2_ Isotopic Analyzer, Picarro, Sunnyvale, CA, USA). After measuring the baseline isotopic enrichment of exhaled breath over a period of 5 min, a bat was taken out of the chamber and injected intraperitoneally with a 170 mg isotonic dosage of ^13^C-labelled sodium bicarbonate (0.29 mol L^−1^, Euriso-Top) and placed back into the respirometry chamber. After complete equilibration, tracer enrichment declined exponentially, at which time we transferred the bat from the respirometry chamber into the wind tunnel, where it flew for 66 ± 4 seconds. After the flight, we returned the bat to the chamber to further record the clearance of the marker for at least 15 min. After the end of trials, all bats were in good health and were thus released at the site of capture.

For analysis of the rate of CO_2_ production (*V̇*co_2_), we converted the *δ^13^*C to atom per cent (AP) according to Slater et al. (2001) and plotted the natural logarithm of excess ^13^C enrichment (ln*AP^13^CE*) in relation to time elapsed since the injection of ^13^C-labeled sodium bicarbonate.

For the pre-flight period, we calculated the fractional isotopic turnover (*k_c_*; min^−1^) according to:

$$k_{c}=\frac{\left[ \mathrm{lnAP}{}^{13}{\mathrm{CE}\left( t_{1} \right)}- \mathrm{lnAP}{}^{13}{\mathrm{CE}\left( t_{2} \right)} \right]}{t_{2}-t_{1}}$$

with ^13^C enrichment above background (*AP^13^CE*) at time *t_1_* (min) and at time *t_2_* (min). We used the peak *AP^13^CE* post-injection to obtain the total body bicarbonate pool, *N*_c_ (mol CO_2_), using the following equation:

$$N_{c}=20 \times e^{(-7.2421-1.5458 \times\ln\mathrm{peak} \mathrm{AP}{}^{13}{C)}}$$

We then multiplied the elimination rate of carbon isotopes, *k_c_*, by the bicarbonate pool of the animal, *N*_c_, and converted this into *V̇*co_2_ (ml min^−1^) using the equation for ideal gases, with the measured barometric pressure and assuming a temperature of 30°C. Plotting the elimination rate (*k*_c_) derived from the bicarbonate method in relation to *V̇*co_2_ from respirometry showed a high precision of the method in resting bats before flight (*R*^2^ = 0.71, *P* < 0.001; Fig S2). For converting *V̇*co_2_ into metabolic power in watts (W), we assumed that bats oxidized predominantly glycogen during the short flight interval and thus we used a conversion factor of 21.1 J per ml CO_2_.

**References:**

Engel, S., Biebach, H., & Visser, G. H. (2006). Metabolic costs of avian flight in relation to flight velocity: a study in Rose Coloured Starlings *Sturnus roseus*, Linnaeus). *Journal of Comparative Physiology B-Biochemical Systems and Environmental Physiology*, *176*(5), 415-427. <https://doi.org/10.1007/s00360-006-0063-1>

Hambly, C., & Voigt, C. C. (2011). Measuring energy expenditure in birds using bolus injections of 13C-labelled Na-bicarbonate. *Comparative Biochemistry and Physiology Part A: Molecular & Integrative Physiology*, *158*(3), 323-328. <https://doi.org/https://doi.org/10.1016/j.cbpa.2010.05.012>

Slater, C., Preston, T., & Weaver, L. T. (2001). Stable isotopes and the international system of units. *Rapid Communications in Mass Spectrometry*, *15*(15), 1270-1273. <https://doi.org/https://doi.org/10.1002/rcm.328>


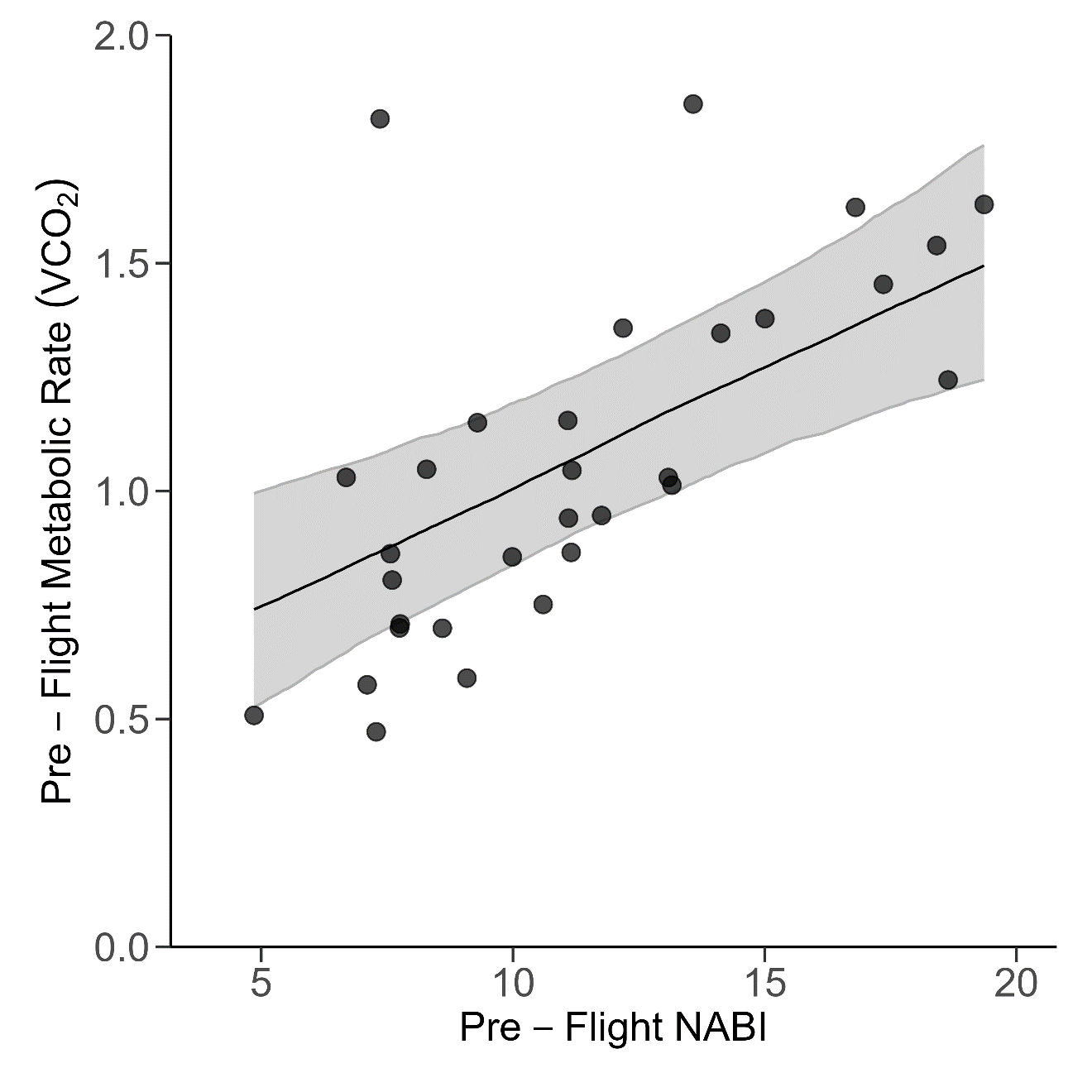


FIGURE S2 Correlation between CO_2_ production rate during the pre-flight period measured by conventional respirometry, i.e. CO_2_ analyser, and by the ^13^C labelled Na-bicarbonate method (NABI)

Table S2 Number of trials per individual common noctule bat flying in the wind tunnel

| Bat ID | Number of trials |
| --- | --- |
| Nnoc03 | 1 |
| Nnoc04 | 1 |
| Nnoc05 | 2 |
| Nnoc06 | 3 |
| Nnoc07 | 2 |
| Nnoc10 | 2 |
| Nnoc11 | 1 |

**Master table of all tracking studies included in the meta-analysis**

All studies complied with national animal welfare and conservation laws. The studies included in our meta-analysis took place in northern and northeastern Germany. The study sites consisted of mostly flat landscapes with either intensive agriculture or silviculture (Roeleke et al. 2016, 2018, 2020, 2022, Reusch et al. 2023). One study took place in the Berlin metropolitan area (Voigt et al. 2020), one in a rural landscape near the North Sea coast in Lower Saxony (Reusch et al. 2022), and one in a suburban area in proximity to Rostock in Mecklenburg-Western Pomerania (Stidsholt et al., 2024). The study by Lagerveld (2023) was considered, but had to be discarded due to inconsistent tracking data and therefore incompatible data. As our approach was based on comprehensive movement data, we neglected radio-tracking studies, assuming that the accuracy of spatial positions would be compromised by the rapid flight of this bat species when using Very High Frequency (VHF) radio transmitters.

Table S3 Meta data and properties of tags used in the meta-analysis. Tag mass is defined as the mass of the whole logger unit, including sometimes a VHF tag and a plastic backpack to protect the unit from water.

| ^­^ | ^Season^ | ^Males^ | ^Females^ | ^Mean relative^  ^tag mass [%]^ | ^Mean^  ^tag mass [g]^ | ^main habitat^ | ^tag type^ | ^Source^ |
| --- | --- | --- | --- | --- | --- | --- | --- | --- |
| ^2014^ | ^May - July^ | ^5^ | ^3^ | ^11.0^ | ^3.7^ | ^farmland^ | ^Robin CellGuide, Lucidlogix Technologies Ltd., Kfar Netter, Israel^ | ^Roeleke et al. 2016^ |
| ^2015, 2016^ | ^July^ | ^4^ | ^5^ | ^11.1^ | ^3.4^ | ^forest site^ | ^Robin CellGuide, Lucidlogix Technologies Ltd., Kfar Netter, Israel^ | ^Roeleke et al. 2018^ |
| ^2016 - 2018^ | ^July, August^ | ^14^ | ^13^ | ^15.9^ | ^4.5^ | ^farmland; forest site^ | ^Vesper GPS, ASDevelopments,^  ^Haifa, Israel^ | ^Roeleke et al. 2020^ |
| ^2015 - 2018^ | ^June - September^ | ^44^ | ^3^ | ^16.1^ | ^4.8^ | ^urban area^ | ^Robin, CellGuide, Ltd., Tel Aviv, Israel;^  ^Vesper, ASD technologies inc., Haifa, Israel^ | ^Voigt et al. 2020^ |
| ^2020^ | ^August^ | ^6^ | ^5^ | ^11.6^ | ^3.7^ | ^farmland^ | ^Vesper GPS, ASDevelopments,^  ^Haifa, Israel^ | ^Reusch et al. 2022^ |
| ^2018 - 2020^ | ^May, July, August^ | ^11^ | ^56^ | ^4.3^ | ^1.2^ | ^farmland^ | ^ATLAS, TAU, Tel Aviv, Israel^ | ^Roeleke et al. 2022^ |
| ^2019, 2020^ | ^May, June, August^ | ^42^ | ^18^ | ^7.9^ | ^2.3^ | ^forest site^ | ^nanoFix GEO-MINI. Pathtrack^  ^Otley, United Kingdom^ | ^Reusch et al. 2023^ |
| ^2020^ | ^June^ | ^4^ |  | ^14.8^ | ^3.8^ | ^urban^ | ^Vesper GPS, ASDevelopments,^  ^Haifa, Israel^ | ^Stidsholt et al. 2023^ |
| ^2019^ | ^July^ |  | ^1^ | ^5.8^ | ^1.6^ | ^farmland^ | ^nanoFix GEO-MINI. Pathtrack,^  ^Otley, United Kingdom^ | ^Reusch, unpublished^ |
|  |  | ^∑ 130^ | ^∑ 104^ | ^⌀ 8.8^ | ^⌀ 2.6^ |  |  |  |
